# The Evolution of the Use of Extracorporeal Membrane Oxygenation in Respiratory Failure

**DOI:** 10.3390/membranes11070491

**Published:** 2021-06-30

**Authors:** Danielle Feldhaus, Daniel Brodie, Philippe Lemaitre, Joshua Sonett, Cara Agerstrand

**Affiliations:** 1 Department of Surgery, New York-Presbyterian Hospital, Columbia University College of Physicians and Surgeons, New York, NY 10065, USA; phl2144@cumc.columbia.edu (P.L.); js2106@cumc.columbia.edu (J.S.); 2 Department of Medicine, New York-Presbyterian Hospital, Columbia University College of Physicians and Surgeons, New York, NY 10065, USA; hdb5@cumc.columbia.edu

**Keywords:** extracorporeal membrane oxygenation, acute respiratory distress syndrome, H1N1 influenza A, coronavirus disease 2019

## Abstract

Extracorporeal membrane oxygenation (ECMO) has been used with increasing frequency to support patients with acute respiratory failure, most commonly, and severe forms of acute respiratory distress syndrome (ARDS). The marked increase in the global use of ECMO followed the publication of a large randomized trial in 2009 and the experience garnered during the 2009 influenza A (H1N1) pandemic, and has been further supported by the release of a large, randomized clinical trial in 2018, confirming a benefit from using ECMO in patients with severe ARDS. Despite a rapid expansion of ECMO-related publications, optimal management of patients receiving ECMO, in terms of patient selection, ventilator management, anticoagulation, and transfusion strategies, is evolving. Most recently, ECMO is being utilized for an expanding variety of conditions, including for cases of severe pulmonary or cardiac failure from coronavirus disease 2019 (COVID-19). This review evaluates modern evidence for ECMO for respiratory failure and the current challenges in the field.

## 1. Background on ECMO for ARDS

Extracorporeal membrane oxygenation (ECMO) was first successfully used for adult patients with severe respiratory failure in the early 1970s; however, broader application was limited by high complication rates [1,2]. Despite poor early outcomes, over the ensuing decades, ECMO continued to be used sparingly at select centers globally. During this time, ECMO circuitry improved in terms of safety, durability, and biocompatibility, such that it was associated with fewer complications and improved clinical outcomes, albeit typically reported in case reports or small case series [3]. In concert with advances in ECMO technology was an improved understanding of the pathophysiology and management of patients with acute respiratory distress syndrome (ARDS), which, apart from ECMO, resulted in improved survival [4].

## 2. ECMO in the Modern Era

The modern era of ECMO support may reasonably be pegged to 2009, when global use increased markedly following the experience during the influenza A (H1N1) pandemic and the publication of the efficacy and economic assessment of conventional ventilatory support versus extracorporeal membrane oxygenation for severe adult respiratory failure (CESAR) trial [5,6,7,8]. The Australia and New Zealand ECMO Investigators (ANZ ECMO) published their experience with ECMO during the H1N1 pandemic and showed a 75% hospital survival rate in patients supported with ECMO [6]. Though the study was observational, thereby lacking a control group with conventional ventilatory management, and the population was relatively young (median age 34.4 years), the high rate of survival despite a high severity of illness (including a median pre-cannulation partial pressure of arterial oxygen [PaO_2_] of 56 mm Hg and positive end-expiratory pressure [PEEP] of 18 cm H_2_O), suggested a potential benefit from ECMO in this population [7].

A systematic review and meta-analysis from the H1N1 pandemic also found positive outcomes with ECMO with an in-hospital mortality of 28% [9]. Similar to the ANZ experience, these patients had a relatively low median age of 36 years and required ECMO for a median of ten days [9]. Additional support for the use of ECMO for influenza A (H1N1) came from a study in which ECMO-referred patients were matched according to demographics, physiologic data, and comorbidities with patients who did not receive ECMO [8]. Multiple matching strategies suggested an approximate 50% reduction in mortality in the ECMO-referred cohort [8]. 

Coinciding with the H1N1 pandemic was the publication of the CESAR trial, the first modern randomized clinical trial of patients supported with ECMO. CESAR randomized 180 adults with severe, early acute respiratory failure (Murray score > 3, pH < 7.2, endotracheally intubated 7 days or less) to conventional ventilatory management or referral to an ECMO center with consideration for ECMO [5]. CESAR showed a significant improvement in six-month survival without severe disability in the ECMO-referred group (63% versus 47%, *p* = 0.03) [5]. However, the pragmatic nature of the CESAR trial meant it was difficult to separate the benefit of ECMO from the benefit of being managed at an experienced tertiary care center, which included greater adherence to lung protective ventilatory strategies [5]. Since the positive results of this trial may be in part due to management at an experienced tertiary care center with lung protective ventilatory strategies, transfer to an ECMO referral center may be considered for critically ill patients (particularly at centers unable to provide adjuvant therapies such as prone positioning or neuromuscular blockade) [5].

A second modern randomized controlled trial for ECMO was designed to further investigate the benefit of ECMO in severe ARDS and address the limitations inherent to the CESAR trial. The extracorporeal membrane oxygenation for severe acute respiratory distress syndrome (EOLIA) trial randomized patients 18–70 years old with early ARDS intubated for fewer than seven days to early ECMO or conventional standard of care mechanical ventilation with a lung protective ventilation strategy [10]. Adjunctive measures such as prone positioning and use of neuromuscular blocking agents were encouraged in both groups. Although the trial was stopped early for futility to reach a 20% reduction in mortality in the ECMO-supported group, it demonstrated an impressive 11% reduction in mortality (*p* = 0.09) and met key secondary endpoints, including a decrease in mortality or treatment failure (which included death or crossover from the standard of care group to the ECMO group) [10]. It is important to note that considerable crossover from the control group to the ECMO group occurred (28% of control subjects) in many of the sickest patients in that cohort, which biased results to the null [10]. Importantly, ECMO was generally well-tolerated. While there were more bleeding events leading to transfusion and occurrences of severe thrombocytopenia in the ECMO-supported group, other complications were similar, including the rate of ischemic or hemorrhagic strokes.

A subsequent systematic review and meta-analysis as well as post-hoc Bayesian analysis of the data from the EOLIA trial suggested a benefit of ECMO in ARDS [11,12]. The Bayesian analysis indicated that the probability of any mortality benefit with early ECMO is high, ranging from 88–99%, depending on one’s priors [11]. An individual patient data meta-analysis evaluated data from the CESAR and EOLIA trials and found a significantly lower 60 day mortality in the venovenous ECMO group compared to the control group (*p* = 0.008) [12]. This analysis also found higher rates of major bleeding in the ECMO group [12]. 

## 3. Criteria for ECMO

The EOLIA criteria has been used as guidance for determining which patients may benefit from ECMO. These criteria include a ratio of PaO_2_ to fraction of inspired oxygen (FiO_2_) of <50 mm Hg for >3 h, PaO_2_-to-FiO_2_ < 80 mm Hg for >6 h, or arterial blood pH < 7.25 with partial pressure of arterial carbon dioxide of at least 60 mm Hg for >6 h, *despite* optimizing mechanical ventilation [10]. 

**Figure 1:**
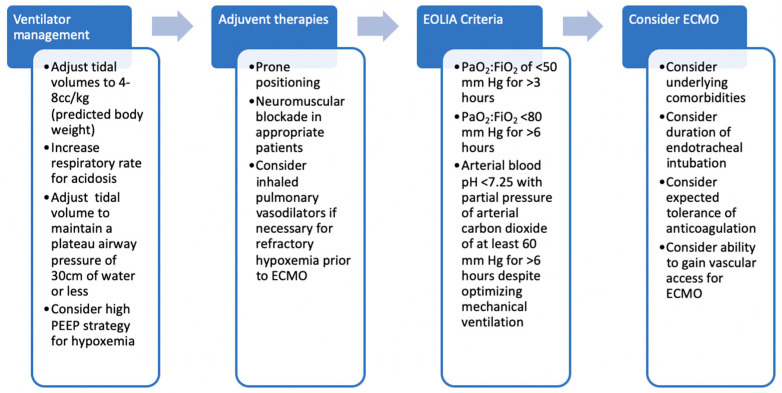
Algorithm for determination of candidacy for ECMO for ARDS. ECMO is appropriate for patients meeting EOLIA criteria despite optimal ventilator management and adjuvant therapies. Abbreviations: ECMO, extracorporeal membrane oxygenation; FiO_2_, fraction of inspired oxygen; PaO_2_, partial pressure of arterial oxygen; PEEP, positive end-expiratory pressure.

This criteria is applied after failure of conventional management as described in Figure 1 [13]. ECMO may also be used as a “rescue” therapy when conventional, evidence-based therapies (such as prone positioning) are contraindicated or unavailable in order to transport patients to an expert center capable of providing specialized therapies, such as transplantation [14]. The only absolute contraindication for ECMO for respiratory failure is an irreversible underlying process when the patient is not a candidate for lung transplantation [14]. Relative contraindications include untreatable metastatic cancer, devastating neurologic injury, intolerance of anticoagulation, and difficult vascular access among others [14]. The likelihood of recovery should also be considered when determining which patients are candidates for ECMO. Factors such as underlying, comorbid disease and number and severity of other organ failures should be considered when determining the appropriateness of ECMO [15].

## 4. Complications

There are many potential complications with ECMO, including medical and device complications. The most common complications reported by the Extracorporeal Life Support Organization (ELSO) International Summary (2016–2020) include: hemorrhage (20.3%), renal replacement therapy (26.9%), and mechanical complications (34.5%) including thrombosis and air emboli, oxygenator or pump failure, and circuit change [16]. The most common hemorrhagic complications reported were surgical site bleeding and GI hemorrhage [16]. Anticoagulation is often used to maintain circuit patency and reduce the risk of thrombosis, but must be balanced against the risk of bleeding. 

## 5. Consideration of Extracorporeal Carbon Dioxide Removal (ECCO_2_R) in Moderate ARDS

ECMO can be used in severe ARDS to support both oxygenation and carbon dioxide (CO_2_) removal. However, in less severe forms of ARDS, extracorporeal carbon dioxide removal (ECCO_2_R) has been utilized to remove CO_2_ at rates of blood flow not sufficient to provide substantial oxygenation support. The use of ECCO_2_R in ARDS is appealing as a mechanism for facilitating lung protective ventilatory strategies that may otherwise be limited by respiratory acidosis [16]. However, though ECCO_2_R has been used successfully in acute exacerbations of chronic obstructive pulmonary disease, severe asthma, and as a bridge to lung transplantation, evidence in ARDS is lacking and its use investigational. [17,18,19,20].

## 6. Ventilator Management

The benefit provided from ECMO may in part be due to its ability to facilitate ventilator settings that may prevent or attenuate ventilator-induced lung injury [14,21,22,23]. The approach to mechanical ventilation during ECMO should target a lower respiratory rate, tidal volume, and airway pressure than would be possible without the concomitant use of ECMO [10,21]. While specific ventilatory strategies vary among centers, in the absence of randomized trials evaluating specific ultra-lung protective ventilation strategies, the approach used in EOLIA is a reasonable strategy for ECMO-supported patients with ARDS. EOLIA targeted a respiratory rate of 10 to 30 breaths per minute, a maximum end-inspiratory plateau airway pressure of 24 cm H_2_O, PEEP ≥ 10, and FiO_2_ 30–50% [10,22]. It has been suggested that lower settings could potentially be beneficial, particularly in terms of a respiratory rate [22]. 

## 7. Anticoagulation

In order to reduce the risk of thrombus formation and maintain circuit patency, in most cases, patients receiving ECMO receive a continuous infusion of systemic anticoagulation, typically unfractionated heparin [24]. However, there has been interest in the use of direct thrombin inhibitors [15]. Although there is no universally accepted anticoagulation target for ECMO, there is a trend toward lower dose anticoagulation, for instance, activated partial thromboplastic time (aPTT) 40 to 60 s [10,25]. While this approach has been associated with lower bleeding complications and need for transfusions, it must be balanced against a potentially increased risk of thrombosis [10,15,25]. 

## 8. Blood Transfusion

Historically, ECMO patients were transfused to maintain a normal hemoglobin in order to optimize oxygen delivery. While some centers still practice this approach, there has been a trend toward tolerance of a moderate degree of anemia (targeting hemoglobin greater than 7 g/dL), which has proven beneficial in critically ill patients not supported with ECMO [26]. Recent studies have evaluated transfusions for lower hemoglobin targets in ECMO patients as well. A study in patients receiving venovenous ECMO compared an approach of transfusing to maintain a hemoglobin of 8 g/dL versus 10 g/dL and found no difference in survival [27]. The mortality rates were comparable to those reported in the Extracorporeal Life Support Organization (ELSO) registry [27]. An observational study of adult patients receiving venovenous ECMO for ARDS managed with a restrictive transfusion trigger with a hemoglobin of <7 g/dL and a low-dose anticoagulation strategy (aPTT 40 to 60 s) demonstrated lower transfusion requirements and bleeding complications than previously reported in the literature, also with comparable survival [25]. These patients also received auto transfusion of circuit blood during decannulation, and survival rates were comparable to or better than most of those reported in the literature [25].

## 9. Extubation and Mobilization

Select patients supported with ECMO may be appropriate for endotracheal extubation. Though data is limited in ARDS, extubation has been shown to be safe and feasible in patients receiving ECCO_2_R, as well as in patients with status asthmaticus [18,20]. Though this approach in ARDS is less well described, one study evaluated 80 patients with ARDS supported with ECMO, in which 12 were managed in an awake, endotracheally extubated manner [28]. In these patients, extubation was achieved after a median of 10.2 days of ECMO support and early mobilization was utilized. The hospital survival rate in this cohort was 66.7% [28]. One potential benefit of awake ECMO is the ability to minimize the use of sedative medications. However, the use of sedation in patients receiving ECMO for ARDS is controversial and remains an area of active exploration. Additionally, awake ECMO may allow improvement in nutrition and facilitate physical rehabilitation [21,29]. However, many key questions remain to be answered about this strategy, particularly in the ARDS population.

## 10. Evolving Applications of ECMO in ARDS

ECMO has also been used for ARDS caused by emerging infectious diseases, such as Middle East respiratory syndrome (MERS) and most recently, coronavirus disease 2019 (COVID-19). MERS was first reported in Saudi Arabia in 2012 and was associated with significant cardiopulmonary disease, with an estimated 6% of afflicted patients receiving ECMO support [29]. Though limited evidence exists on the use of ECMO in MERS, one retrospective cohort study evaluated 17 patients receiving ECMO and 18 patients receiving conventional therapy in Saudi Arabia for MERS [30]. The ECMO group had significantly lower in-hospital mortality (65 vs. 100%, *p* = 0.02) [30]. 

The World Health Organization characterized COVID-19 as a pandemic on 11 March 2020 [31]. Early in the pandemic, ECMO was used in China for patients with COVID-19, but studies were limited and its benefit uncertain [32]. In addition to unclear benefit, resource allocation was a concern, particularly in viral hotspots [33,34]. As the pandemic has progressed, more data has become available. A report from the of ELSO registry data showed greater than 60% survival in patients with primarily ARDS secondary to COVID-19 [35]. However, patient selection is critical, as those with advanced age or other comorbidities may have inferior outcomes [35,36,37,38]. At this point, these data suggest that ECMO should be considered as part of a management protocol for patients with severe ARDS secondary to COVID-19; however, as global experience with ECMO for patients with COVID-19 grows and as outcomes evolve, the calculus for considering ECMO in these patients may change over time.

## 11. Conclusions

ECMO is increasingly being used for severe cases of ARDS. The use of ECMO during the 2009 influenza A (H1N1) pandemic, the publication of the CESAR and EOLIA trials, and most recently, the use of ECMO during the COVID-19 pandemic have contributed to the increased adoption of this technology and its incorporation into ARDS management algorithms. As evidence regarding optimal patient selection, timing of ECMO and management of ECMO-supported patients is expanded and refined, clinical practices will continue to evolve, with the hope of optimizing use of this potentially life-saving technology. 

## Data Availability

Not applicable.
